# The potential of TOF PET-MRI for reducing artifacts in PET images

**DOI:** 10.1186/2197-7364-2-S1-A77

**Published:** 2015-05-18

**Authors:** Andrei Iagaru, Ryogo Minamimoto, Craig Levin, Amid Barkhodari, Mehran Jamali, Dawn Holley, Zaharchuk Greg

**Affiliations:** Stanford University, USA

Here we evaluated the potential of TOF PET/MRI to reduce various PET image artifacts, by comparing the images to non-TOF PET/MRI, TOF PET/CT and non-TOF PET/CT.

## Methods and materials

All patients underwent a single-injection of FDG, followed first by PET-CT and subsequently by PET-MRI scan. The PET/CT exams were performed using a GE Discovery 690 PET/CT scanner. The PET/MRI images were acquired on a GE Signa PET/MRI scanner. All PET images were reconstructed with and without the TOF data. Visual analysis of these series was performed for dental metal / breathing artifcats and 3) artifacts caused by high excretion of FDG in the bladder. PET image quality was evaluated using a 3-point scale (1 - clinically significant artifact; 2 – non clinically-significant artifact; and 3 - no artifact). Data from 18 oncologic patients (mean ± SD age: 55 ± 10 years; female 7, male 11) were used. The average scores of TOF PET/MRI, non-TOF PET/MRI, TOF PET/CT and non-TOF PET/CT for dental artifacts were 3.0, 2.8, 2.4 and 2.3, respectively; for breathing artifacts were 3.0, 2.5, 2.5 and 2.3, respectively; and for pelvic artifacts were 2.9, 1.6, 2.1 and 1.4, respectively. TOF PET/MRI had the highest image quality scores among the 4 series of PET data analyzed for these types of artifacts. TOF PET/MRI showed promising results in reduction of various PET artifacts in this cohort, when compared to non-TOF PET/MRI, TOF PET/CT and non-TOF PET/CT. This may be due to the better timing resolution (<400 ps) for the PET/MR system compared to the PET/CT system (>600 ps).

